# The Random Step Method for Measuring the Point of Subjective Equality

**DOI:** 10.3390/vision7040074

**Published:** 2023-11-15

**Authors:** Penghan Wang, Alexandre Reynaud

**Affiliations:** McGill Vision Research Unit, Department of Ophthalmology & Visual Sciences, McGill University, Montreal, QC H3G 1A4, Canada; penghan.wang@mail.mcgill.ca

**Keywords:** psychophysics, point of subjective equality, PSE, adaptive method, psychometric function

## Abstract

Points of Subjective Equality (PSE) are commonly measured using staircase or constant stimuli methods. However, the staircase method is highly dependent on the step size, and the constant stimuli method is time-consuming. Thus, we wanted to develop an efficient and quick method to estimate both the PSE and the slope of the psychometric function. We developed a random-step algorithm in which a one-up-one-down rule is followed but with a random step size in a pre-defined range of test levels. Each stimulus would be chosen depending on the previous response of the subject. If the subject responded “up”, any random level in the lower range would be picked for the next trial. And if the subject responded “down”, any random level in the upper range would be picked for the next trial. This procedure would result in a bell-shaped distribution of the test levels around the estimated PSE, while a substantial amount of trials would still be dispersed at both bounds of the range. We then compared this method with traditional constant stimuli procedure on a task based on the Pulfrich phenomenon while the PSEs of participants could be varied using different neutral density filters. Our random-step method provided robust estimates of both the PSE and the slope under various noise levels with small trial counts, and we observed a significant correlation between the PSEs obtained with the two methods. The random-step method is an efficient way to measure the full psychometric function when testing time is critical, such as in clinical settings.

## 1. Introduction

One common measurement in psychophysical studies is the point of subjective equality or PSE. PSE can be described as the point where a test stimulus subjectively looks like a standard stimulus to one observer who would choose randomly between them [1] Several methods can be used to measure a subject’s PSE. The standard constant stimuli (CS) method uses the same number of repetitions at each level [2,3,4]. It can obtain low-variability PSE estimates and is quite robust when the trial number is low [4]. However, it may underperform when effects of adaptation, habituation, and sensitization occur [5] and can be quite time-consuming [6], thereby not well-suited in clinical settings.

Therefore, several faster adaptive methods have been developed for the measurement of PSEs or thresholds. Instead of fixing the stimulus levels and the number of repetitions before the experiment, the values will depend on one or several preceding trials [7]. The most common adaptive method is the staircase (SC) procedure. With this method, stimuli are presented in ascending and descending levels order. When the observer’s response changes, the direction of the stimulus sequence will be reversed. So, unlike the CS method, the SC method guarantees to centralize almost all the trials around the PSE for a precise estimate. It is an efficient, powerful method for PSE measurement because, as the algorithm progresses, the tested stimuli would be chosen around the PSE estimate [8]. Similar adaptive up/down methods were proposed as well, aiming to cue the magnitude of the stimulus on the next trial, with different rules to determine the step size [9]. However, it may not provide an accurate estimate of the slope of the psychometric function due to its dependence on step size and the concentration of samples around the PSE [10]. The slope of the psychometric function is more of a rate of change in the observer performance in psychophysics studies. It is used to test sensitivity to specific stimuli [11] and can reflect the relative noise level during an experiment [12,13].

In order to have a more appropriate and comprehensive display of psychometric functions, both the measurement of location and dispersion should be considered [14]. Hence, other adaptive methods have been developed to optimize the estimation of both the PSE and the slope of the psychometric function based on maximum likelihood: modified Best PEST [15,16] and Bayesian estimation: qCD [17,18], qVFM [19]. Bayesian methods take advantage of prior information from subjects to guide the placement of trials [20,21] for estimating the psychometric function. Concretely, on a trial-by-trial basis, they use complex algorithms to determine the optimal stimulus level to test to gain the most information [7,16]. All of those adaptive methods are very efficient and are able to inference parameters values estimates and confidence intervals of the psychometric function accurately [15]. But they may also present low versatility [22]. For example, as a parametric method, the qCD method needs to tune the specifics of the implementation in each case to guarantee the posterior convergence rate [17]. In addition, the qVFM method is mostly designed for estimating visual function maps of individuals at the moment [19]. 

As a result, in this study, we set out to design a robust, simple, and accurate measurement of PSE with an accompanying accurate estimate of the slope of the psychometric function. Our novel Random Step (RS) method chooses each stimulus in a pre-defined range of test levels. It follows a one-up-one-down rule but with a random step size: at each trial, any level on a given side of the previous test level, depending on the subject’s response, can be picked. To verify the functioning of the RS method, we first compared it to standard constant stimuli and staircase procedures in a series of simulations. 

Then, we analyzed the results from an actual psychophysical experiment by applying it to a paradigm based on the Pulfrich phenomenon [23] that we used in our previous studies [24]. Subjects were presented with an illusory cylinder rotating in depth. The cylinder is defined by oscillating Gabor patches that are presented with an interocular phase shift. A positive phase difference leads to a perceived clockwise rotation, and a negative phase difference leads to an anticlockwise rotation. This task provides a good framework to test the distribution of trials around the PSE because the perceived direction of rotation can be altered by putting a neutral-density (ND) filter in front of one eye, thereby shifting the PSE of ambiguous motion direction as a function of the interocular phase.

## 2. Materials and Methods

### 2.1. Participants

Four subjects (3 males, mean age = 26.8±7.7 years, two authors) with normal or corrected-to-normal vision participated in the main experiment of the study. For the control experiment, Subject 4 (female) was replaced by another subject (female) due to failed contact. Subjects were allowed to take breaks between sessions, and the overall experiment was spread over several days to prevent fatigue effects. This research has been approved by the Ethics Review Board of the McGill University Health Center and was performed in accordance with the ethical standards laid down in the Code of Ethics of the World Medical Association (Declaration of Helsinki). Informed consent was obtained from all subjects.

### 2.2. Apparatus

The experiments were programmed in MATLAB 2015a (© the MathWorks, Natick, MA, USA) with Psychtoolbox-3 extension [25]. In the main experiment, tasks were run on an Apple Mac Pro with an Nvidia GeForce GT graphics card. In the control experiment, tasks were run on an ASUS Zenbook laptop with an AMD Radeon graphics card. In both cases, a wide 23″ ViewSonic V3D231 LED monitor was used to present dichoptic stimuli. The screen was set to a mean luminance of $100\;\mathrm{cd}/m^{2}$ gamma corrected at a resolution of 1920×1080 px, and a refresh rate of 60 Hz. Subjects were required to sit in a dim-lit room at a 90 cm distance from the screen. Subjects wore passive polarized 3D glasses with or without a neutral density (ND) filter (optical density = 1) in front of one of their eyes. The glasses could reduce about 60% of the luminance and generate a 1% crosstalk.

### 2.3. Algorithm

**Figure 1:**
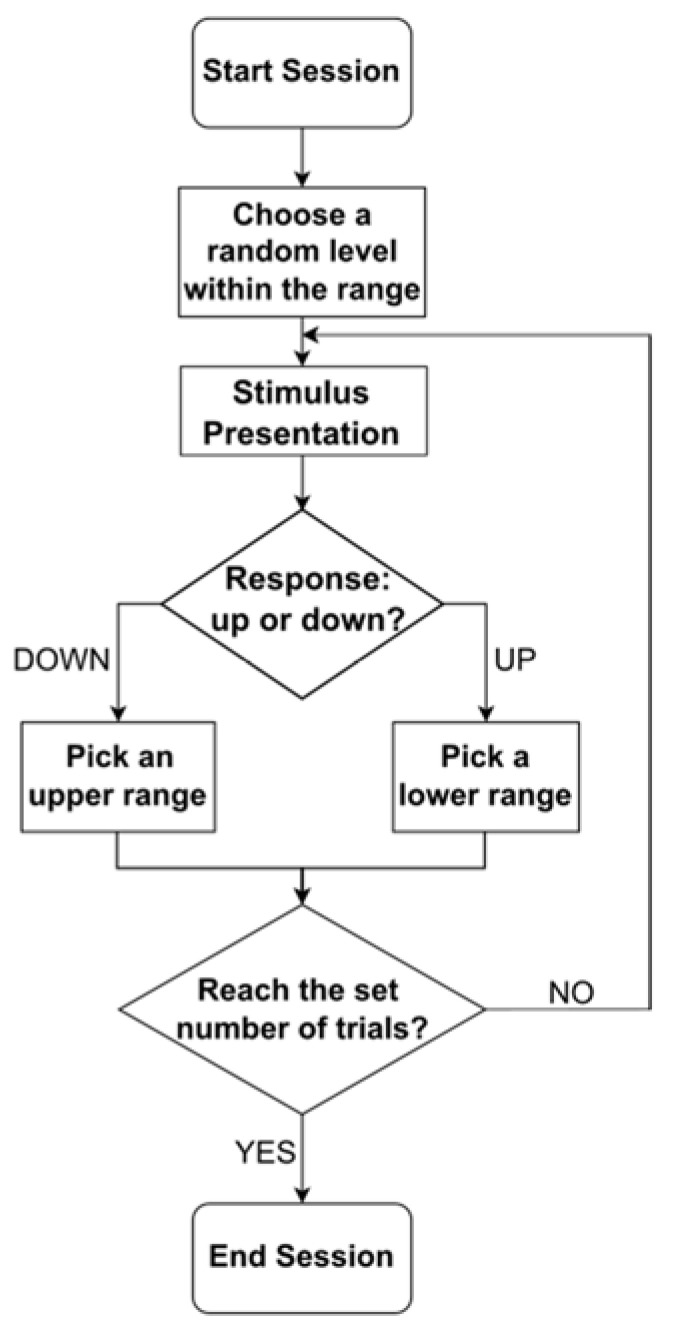
Flowchart of the Random Step method.

The random step (RS) method followed a staircase procedure with a one-up-one-down rule but with random step sizes in a pre-defined range of test levels (Figure 1). First, an initial level would be randomly chosen. The stimulus would be presented, and after the stimulus presentation, the subject would respond “up” or “down” in a 2-alternative forced choice fashion according to their perception. If the subject responded “up”, the next level would be picked anywhere in the range below the tested stimulus, according to a flat distribution. If the subject responded “down”, the next level would be picked anywhere in the range above the tested stimulus. The procedure would be repeated multiple times until the set number of trials was reached. In the algorithm description, “up” and “down” responses were used to describe values above or below the PSE, as it is the standard in such algorithms. In the case of our Pulfrich experiment, “up” and “down” actually mapped to “clockwise” and “anticlockwise” responses (see Section 2.5).

Notably, the range that we chose a level within would never be narrowed down or partially excluded. Unlike the standard staircase method [8], there was always a chance that the stimulus would be picked anywhere within the whole range, even at boundaries.

### 2.4. Stimuli

**Figure 2:**
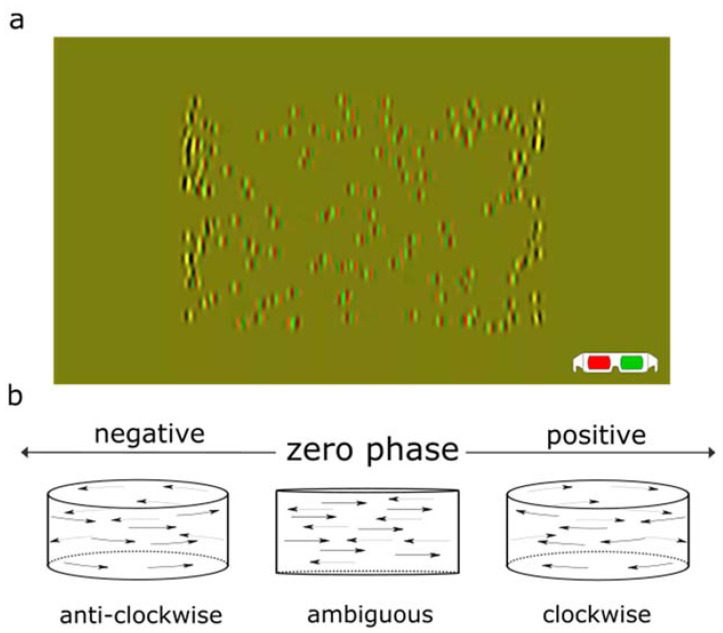
(**a**) Anaglyph representation of the stimulus (for representation only, the actual stimulus was presented using a polarized passive 3D screen). It consists of 200 Gabor patches that are oscillating horizontally and presented dichoptically. As an interocular phase shift is introduced, the stimulus is seen as a rotating cylinder in depth using 3D glasses. (**b**) Schematic illustration of the direction of rotation of the cylinder. The phase difference in the oscillation of the Gabor patches between the eyes generates different percepts of the rotating direction of the motion-defined cylinder in depth. When the phase shift is negative, anticlockwise rotation is perceived; when the phase shift is positive, clockwise rotation is perceived; when the phase is null or close to zero, an ambiguous oscillation in the plane is perceived.

The stimuli we used were similar to the ones used in our previous study [24]. A structure-from-motion-defined cylinder rotating in depth was presented. Each stimulus consisted of 200 Gabor patches that were displaced between the two eyes as a function of the interocular phase difference in their oscillation (Figure 2a). The interocular phase shifts were varied in each trial within −1.5, −0.75, −0.375, −0.1875, −0.0938, −0.0469, −0.0234, 0, 0.0234, 0.0469, 0.0938, 0.1875, 0.375, 0.75, and 1.5 degrees. For comparison between experiments and simulations, phase shifts might be displayed as levels from −7 to 7 in the following analyses.

### 2.5. Procedures

In each trial, participants were asked to respond whether the cylinder was seen rotating clockwise or anticlockwise after an 800 ms presentation (Figure 2b) [24]. Participants were tested using both our RS method and the standard CS method. Three sets of numbers of trials were introduced: 75, 150, and 300 trials. Different numbers of repetitions would be implemented at each level depending on the measuring method: a variable number with our RS method as described above or the same number of repetitions with the CS method. Additionally, the experiment was performed under 3 luminance conditions: a 1ND filter in front of the left eye, no filter, and a 1ND filter in front of the right eye. These three were denoted as −1ND, 0ND, and 1ND, respectively. All 18 experimental conditions (2 methods × 3 sets of trial amount × 3 ND filters) were randomly shuffled during testing. 

In the control experiment, the procedures were almost the same as above, apart from the smaller number of trials: 15, 30, and 45 trials. In addition, instead of 2 methods, 3 methods (RS, CS, and one-up-one-down staircase (SC) method) were performed solely in this part. A total of 27 experimental conditions (3 methods × 3 sets of trial amount × 3 ND filters) were tested in a randomized sequence.

### 2.6. Data Analysis

The data were analyzed with MATLAB R2021b (© the MathWorks). Psychometric functions were fitted with a logistic function forced between 0 and 1 using the Palamedes Toolbox [9]. The estimated midpoint of the logistic function defined the point of subjective equality (PSE), the point at which the perception was 50% clockwise and 50% anticlockwise. The slope of the psychometric function was also taken into consideration in this study. Standard deviations (SDs) were calculated by bootstrapping. Student’s *t*-test was used for testing significance. Bland–Altman plots were generated by a custom function [26].

## 3. Results

### 3.1. Simulations

We first performed simulations on a large number of trials to verify how the Random Step (RS) method operates and observed the resulting distribution of trials. The conditions simulated were 20 test levels, 50,000 trials, and an expected PSE pivot of 13. At each test level i, a value was drawn from a normal distribution centered on a said test level $N\left(i,\sigma \right)$ with a noise level of σ=2 and compared to the pivot. “Test level” could refer to the level of modulation in any dimension in real experiments, while the “noise level” represented the magnitude of noise in the judgment of the simulation. The next test level was then picked following our RS algorithm (Figure 1).

**Figure 3:**
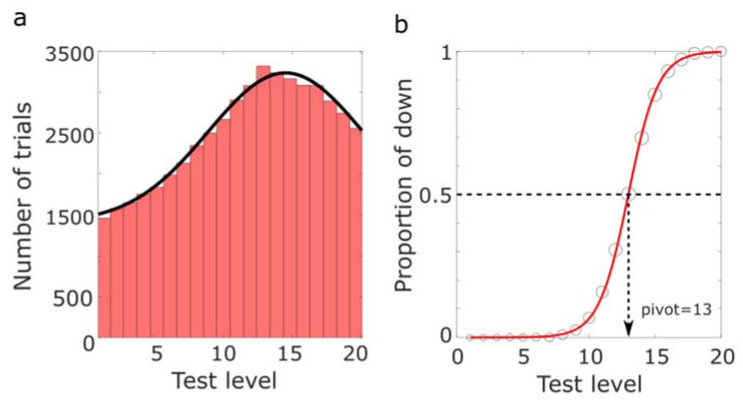
(**a**) The distribution of test levels from the simulation of 50,000 trials with a noise level σ = 2. The black curve represents a Gaussian + offset fit. (**b**) The psychometric function from the simulation is a function of test levels. The size of the circles indicates the number of trials tested at each level.

The results of the simulation are shown in Figure 3. The histogram of the simulated levels in Figure 3a showed that, according to our expectation, with tasks that had a great number of trials, a large proportion of those trials would be dispersed around the PSE estimate while a substantial amount (about half) would still be tested at both bounds of the whole range of test levels. The shape of the distribution was accurately fitted by a Gaussian model with an added vertical offset. The psychometric function resulting from the simulation is shown in Figure 3b. The fact that trials were tested at all levels allows a good fitting and a good estimate of the slope. 

**Figure 4:**
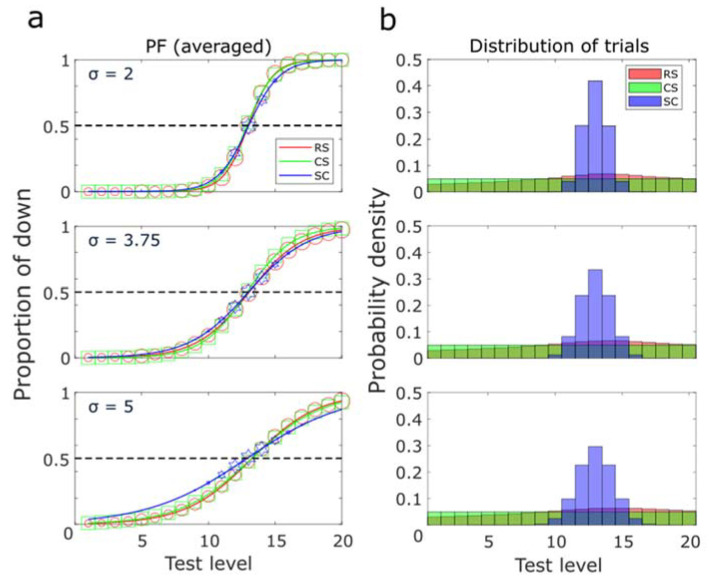
Simulations with random step (RS), constant stimuli (CS), and staircase (SC) methods under different noise levels (2, 3.75, and 5). (**a**) average psychometric functions. RS: red circles; CS: green squares; SC: blue hexagrams. The size of the symbols indicates the number of trials applied at each test level. (**b**) the distribution of trials from the RS, CS, and SC methods in one cycle. In each condition, 10,000 simulation cycles of 100 test trials were implemented with a pivot PSE of 13. SDs were estimated from bootstrapping each of those simulations.

After observing that our RS method could generate accurate psychometric functions, we then compared it with two standard methods: the constant stimuli (CS) method and the staircase (SC) method in a range of more practical conditions (Figure 4). The range of test levels and the PSE pivot remained unchanged. However, the number of trials in a block was decreased to 100 to reflect practical testing conditions. We performed 10,000 repetitions for each 100-trial block simulation with these three different methods to generate distributions of the estimated parameters. Three noise levels (σ = 2, 3.75, or 5) were tested so that we could observe how the methods behave under those different conditions.

As shown in Figure 4a, at each noise level, all psychometric functions had a substantially consistent PSE, and except for noise level 5 with the SC method, all methods yielded very similar psychometric functions. In agreement with earlier studies, as the noise level increased, the slope of the function gradually decreased (the curve became flatter) [11,27,28]. The histograms of the test levels with each method are presented in Figure 4b. The CS method always used the same number of trials at each level and thus showed a flat histogram. The SC method generated a steep trial distribution, which became flatter with an increasing noise level. With our RS method, the distribution of trials became relatively concentrated around the PSE estimate, showing a smooth bump around the pivot but with a fair proportion of trials still dispersed at both bounds. As observed with previous simulations (Figure 3a), here, even with a smaller number of trials, our method tested 2 times more trials around the estimated PSE than at the borders of the range. The distribution from the RS method also became flatter when the noise level increased. With the RS method, the distribution of trials was concentrated around the PSE estimate and reasonably broad for the convenience of calculating the slope of the psychometric function.

**Figure 5:**
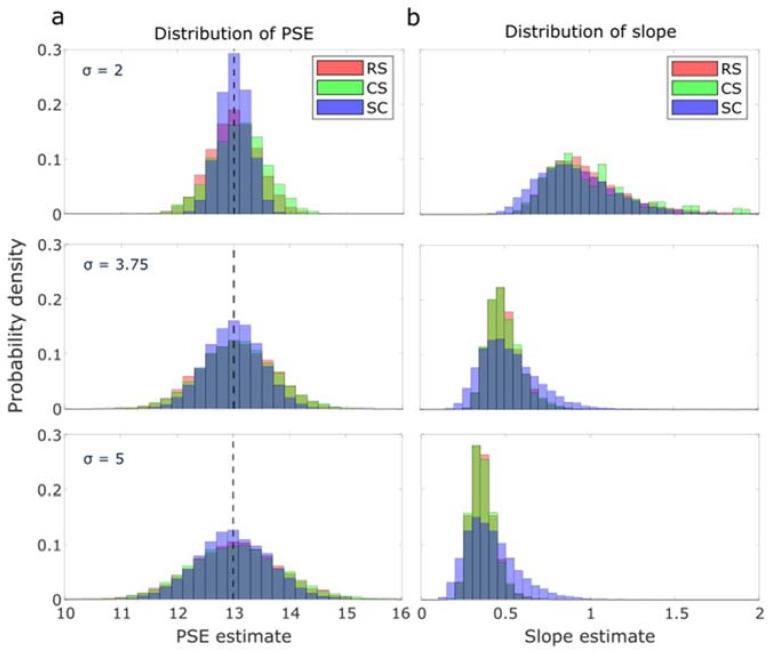
(**a**) normalized histogram of PSE estimates. (**b**) normalized histogram of slope estimates. In each condition, 10,000 simulation cycles of 100 test trials were implemented under different noise levels (2, 3.75, and 5). The expected pivot of PSE was 13, marked out with a black dotted line.

In Figure 5, we presented the distributions for both PSE estimates and slope estimates. Regarding PSE estimates, when considering a ground truth pivot of 13, all distributions were observed to center around the pivot value (Figure 5a), irrespective of the measurement method employed. As the noise level increased, these distributions tended to become wider and flatter. The SC method provided sharper estimates, particularly at low noise levels (σ = 2). The slope estimates (Figure 5b) exhibited dramatic variations with changing noise levels. The distributions shifted to the left, implying smaller means, as the noise level increased. Contrarily to PSE estimates, the SC method provided notably broader histograms, suggesting more variability compared to the two other methods in the slope estimates. 

**Figure 6:**
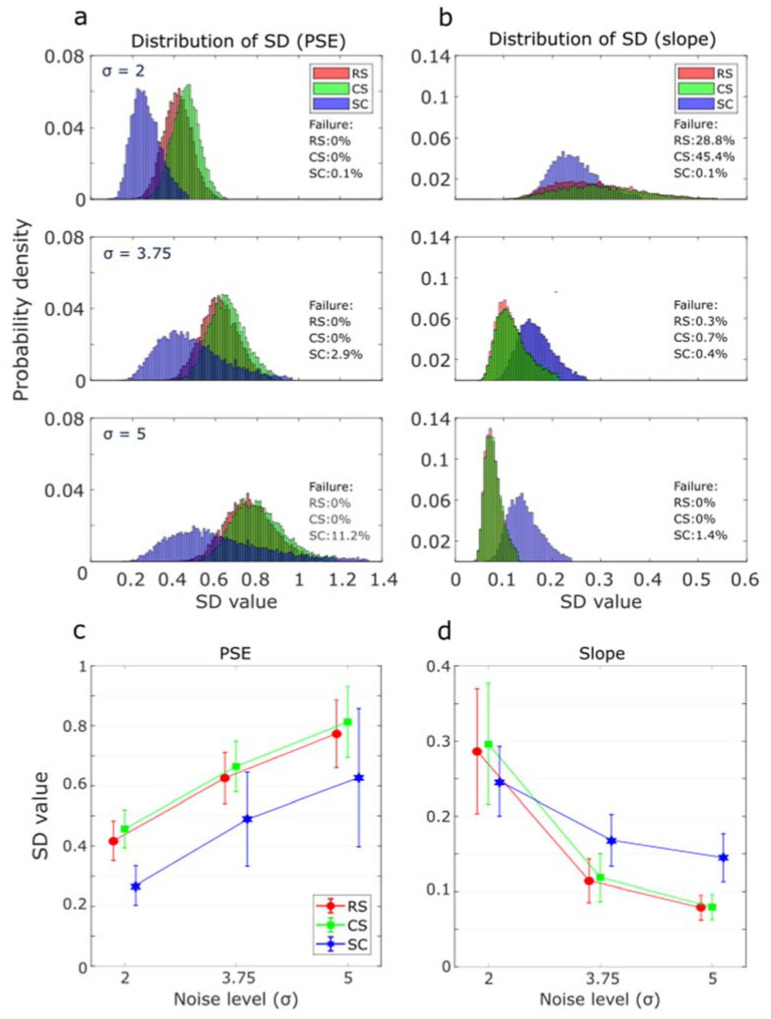
(**a**) normalized histogram of the estimated standard deviations (SD) of the PSE. (**b**) normalized histogram of the estimated standard deviations (SD) of the slope. The failure rates shown on the right side of each histogram refer to invalid SD estimation. The mean of the standard deviation of the PSE (**c**) and slope (**d**) estimated from bootstrapping of the simulations as a function of the noise level. Error bars represent SD.

To check the consistency of those methods, we built distributions of the estimated standard deviations of the PSE and the slope by bootstrapping each of those 10,000 repetitions (resulting in 10,000 estimates of the SD). The histograms of the SDs of the PSE and slope were illustrated in Figure 6a and Figure 6b, respectively. A more precise comparison of the average of those SDs was presented in Figure 6c,d. We can see in Figure 6a that for all methods, the SDs of the PSE shifted to the right and became flatter as the noise level increased. The mean SD from the SC method was always smaller than from the RS and CS methods (see Figure 6c). For the SD distribution of the slope (Figure 6b), on the contrary, as the noise level increased, all distributions shifted to the left and became more concentrated. We can see in Figure 6d that the average SDs from RS and CS were smaller than that from the SC method except when the noise level was 2. The SDs from the RS method were significantly smaller than the CS (p<0.001 for all noise levels). 

Failure rates were computed as the percentage of invalid SD estimations and are presented in each panel. For PSE estimations, failures were only observed with the SC method (Figure 6a), and the failure rate went up with the noise level. This result suggested that the SC method lost robustness in estimating PSE with high noise levels. For slope estimations (Figure 6b), failure rates derived from the SC method were very low for all noise levels. However, it was quite high for both RS and CS methods at a low noise level (σ= 2) and dropped close to 0 when the noise level was increased (σ= 3.75 or 5). Those simulations indicated that our RS method could be well adapted to actual psychophysical testing. So, we then compared its performance to the CS method in a series of experiments. We focused on those two methods because they demonstrated comparable performances in simulations.

### 3.2. Psychophysics

**Figure 7:**
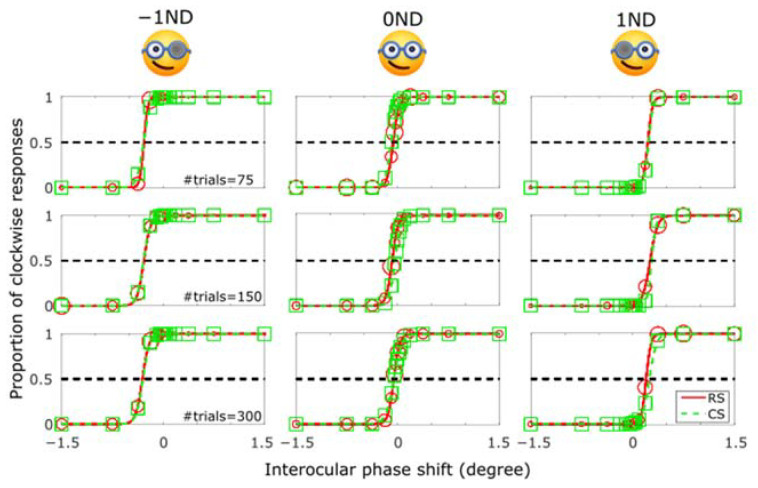
Psychometric functions of one representative subject for the 3 viewing conditions (−1ND, 0ND, and 1ND) using the RS (red) and CS (green) protocols, with 75 (top row), 150 (middle row) and 300 trials (bottom row). The size of the red circles represents the number of trials involved at each phase level in the RS method.

**Figure 8:**
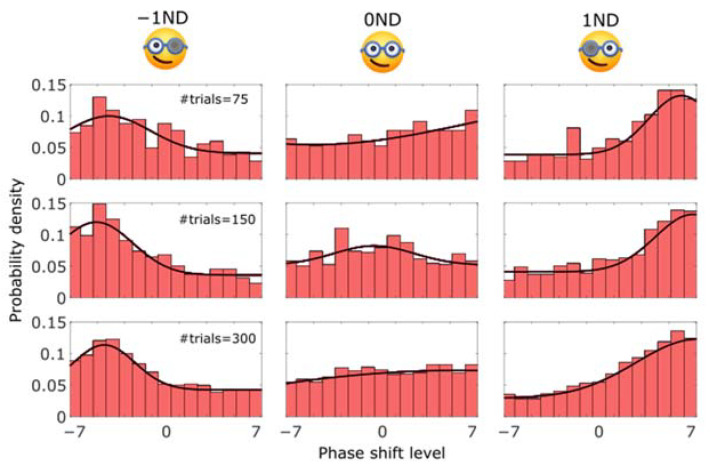
Distribution of the number of trials in the RS method for the different phase shift levels (not isometric) under the 3 viewing conditions (−1ND, 0ND, and 1ND) with 75 (top row), 150 (middle row), and 300 trials (bottom row). These figures were plotted from the same subject’s data in Figure 7. The red curve in each graph represented a Gaussian + offset fit to the distribution.

Next, to assess the validity of our RS method, we compared it to the CS method in an actual psychophysical task based on the Pulfrich phenomenon. We manipulated the luminance seen by each eye by placing a 1ND filter in front of one or none of the eyes (see Methods) to shift the psychometric function [24]. The psychometric functions of one subject under various testing conditions are shown in Figure 7. Under the −1ND condition (filter on the left eye), the curves shifted to the left (negative PSEs); under the 1ND condition (filter on the right eye), the curves shifted to the right (positive PSEs); and under the 0ND condition (no filter) the curves stayed in the middle. The RS and CS methods both accurately fitted the data despite the different sampling strategies, as could be noticed from the size and position of markers (see also histograms in Figure 8). In particular, the differences in the number of trials did not appear to affect the shape of the psychometric functions. 

From the simulations, we expected that the histograms of trials would appear as Gaussian distributions with a vertical offset. Histograms of trial distributions from all conditions in the actual psychophysics experiment are shown in Figure 8. In the −1ND and 1ND conditions, it’s clear that the trials tended to concentrate in one place, with distributions shifted to the left and the right, respectively. The peaks of the distribution were located around the PSEs, as observed in Figure 7, and could themselves provide a good estimate of the PSEs. However, contrary to expectations, for the 0ND condition, the distributions did not show such a clear bell shape around the 0 phase-shift level and tended to be flatter. 

**Figure 9:**
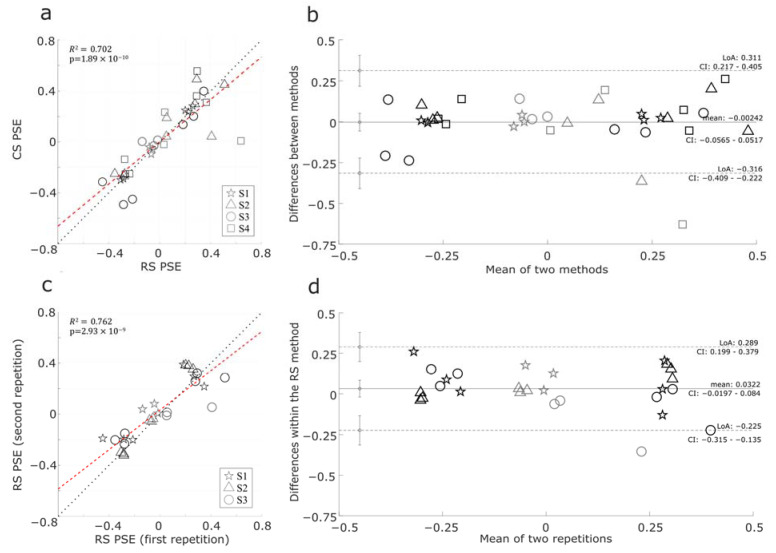
(**a**) correlation of the PSE estimates of 4 subjects with the RS and CS methods. (**b**) the Bland–Altman plot of the data between the RS and CS methods. (**c**) correlation of the PSE estimates of 3 subjects between two repetitions within the RS method. (**d**) the Bland–Altman plot of the data within the RS method. PSE estimates were from the 3 viewing conditions and the 3 sets of numbers of trials tested. Different markers represented individual subjects. Gray markers indicate 0ND condition. The black lines in the scatter maps represent the identity, and the red line represents linear regression.

Despite those differences, the two methods led to comparable PSE estimates. Figure 9a showed a scatterplot of PSEs estimated with the CS and the RS methods in all tested conditions from four subjects, and Figure 9b showed the Bland–Altman plot between the two methods. Roughly, we could observe that the points form three clusters in both figures. In Figure 9a, the lower left, middle, and upper right clusters corresponded to −1ND, 0ND, and 1ND conditions, respectively. The estimates using the two methods were significantly correlated ($R^{2}=0.702$, p<0.001). In the Bland–Altman plot, the mean was close to 0, and the points were evenly distributed around the mean difference line, which indicates that no method consistently yielded higher or lower values.

We also conducted a repeat experiment with our RS method to validate the consistency of the results on a subset of three subjects. Figure 9c,d illustrate scatterplots and Bland–Altman plots comparing the two repetitions with the RS method. Similar to the comparison between the two methods above, three clusters of data points could be found in Figure 9c,d. The estimates between repetitions are slightly more correlated ($R^{2}=0.702$, p<0.001) in Figure 9c, further affirming the reliability of the RS method. And slightly narrower limits of agreement were found between repetitions than the one between methods. These results underscored the consistency of our RS method and its validity compared to CS.

**Figure 10:**
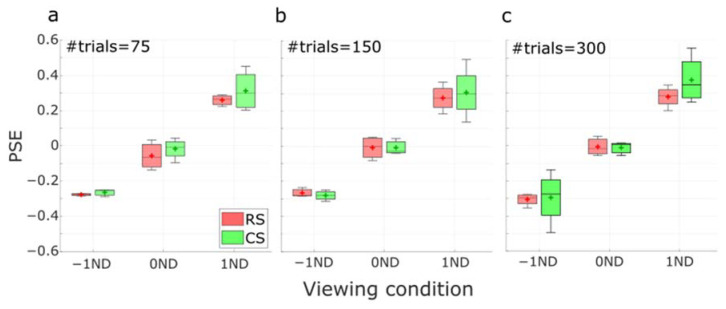
Boxplots of the distribution of PSE estimates using RS and CS protocols as a function of viewing conditions (−1ND, 0ND, and 1ND) at (**a**) 75, (**b**) 150, and (**c**) 300 trials. Horizontal lines and crosses in the boxes represent the median and mean values of the PSE estimates. Error bars represent the standard deviation of the PSE estimates.

After determining the degree of correlation between the PSEs, we next investigated the degree of dispersion of the PSE estimates from the two methods. This would help us understand the performance of the two methods under various conditions. In Figure 10, the distributions of the PSE estimates across subjects for different ND filter positions using different numbers of trials were represented in boxplots. The results were very dependent on the luminance condition. However, the distributions from the RS and CS methods did not present any significant difference between the two methods under all three luminance conditions (p>0.25 in all conditions).

**Figure 11:**
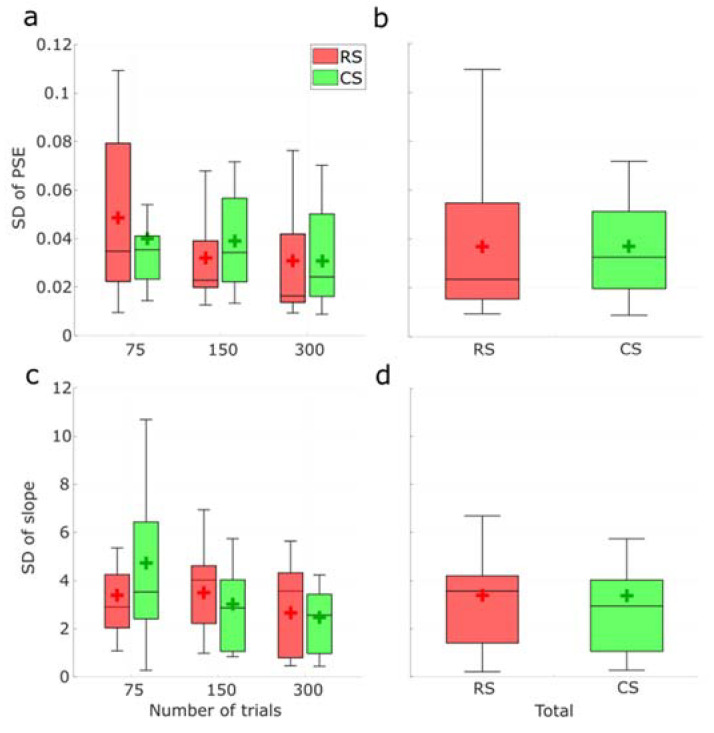
(**a**) Distribution of SDs of the PSE for 75, 150, and 300 trials between RS and CS protocols, respectively. (**b**) Distribution of SDs of the PSE in total between RS and CS protocols. (**c**) Distribution of SDs of the slope for 75, 150, and 300 trials between RS and CS protocols, respectively. (**d**) Distribution of SDs of the slope in total between RS and CS protocols. Horizontal lines and crosses in the boxes represent the median and mean values of PSE estimates, respectively.

Finally, the degree of dispersion was compared according to the standard deviation (SD) of the PSE and slope obtained from bootstrapping of the actual psychometric functions. In Figure 11a, the medians SD were smallest with the RS method for all numbers of trials, and the dispersions were narrower except when the number of trials was 75. When cumulating all number of trial conditions (Figure 11b), the RS method achieved a smaller median SD but a slightly wider dispersion. However, no significant difference was found between those methods in all cases (p>0.3 in all conditions). In terms of slope SDs, smaller median SD and narrower dispersion were only found when the number of trials was 75 (Figure 11c). When combined (Figure 11d), a slightly larger median was observed with the RS method. Again, none of these comparisons was significantly different (p>0.2 in all conditions).

### 3.3. Control Experiment

In order to verify the performance of the RS method in short-run experiments, a control experiment using all three methods (RS, CS, and SC) with a low number of trials, 15, 30, or 45, was implemented. 

**Figure 12:**
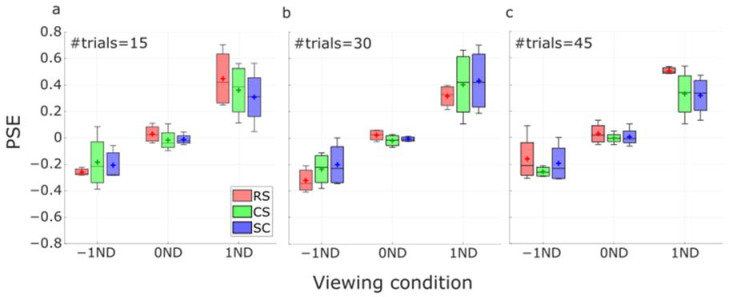
Boxplots of the distribution of PSE estimates using RS, CS, and SC protocols as a function of viewing conditions (−1ND, 0ND, and 1ND) at (**a**) 15, (**b**) 30, and (**c**) 45 trials. Horizontal lines and crosses in the boxes represent the median and mean values of the PSE estimates. Error bars represent the standard deviation of the PSE estimates.

The distributions of the PSE estimates across subjects from different numbers of trials are presented as boxplots in Figure 12. As we can see, the dependence on the luminance conditions remained in short-run tests. An extremely large PSE estimate from the RS method was found under the 1ND condition when the number of trials was 45. However, the distributions from all three methods did not present any significant difference between each other under all three luminance conditions (*p* > 0.2 in all conditions, ANOVA).

**Figure 13:**
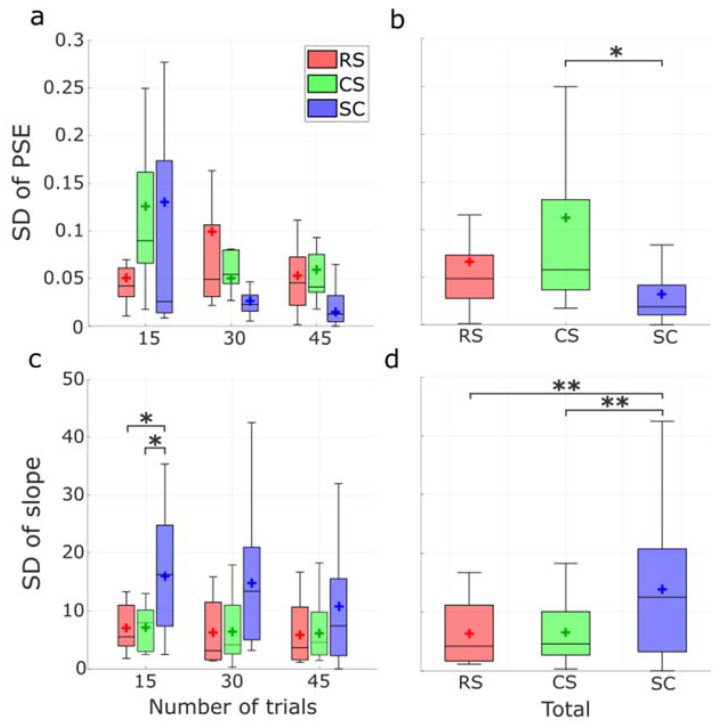
(**a**) Distribution of SDs of the PSE for 15, 30, and 45 trials among RS, CS, and SC protocols, respectively. (**b**) Distribution of SDs of the PSE in total among RS, CS, and SC protocols. (**c**) Distribution of SDs of the slope for 15, 30, and 45 trials among RS, CS, and SC protocols, respectively. (**d**) Distribution of SDs of the slope in total among RS, CS, and SC protocols. Horizontal lines and crosses in the boxes represent the median and mean values of PSE estimates, respectively. P-values less than 0.05 or 0.01 were flagged with one asterisk (*) or 2 asterisks (**), respectively.

The degree of dispersion was then compared according to the standard deviation (SD) of the PSE and slope obtained from bootstrapping of the psychometric functions. In Figure 13a, no significant difference was found among methods in all cases (*p* > 0.1 in all conditions). When cumulating all number of trial conditions (Figure 13b), the RS method achieved a smaller median SD and narrower dispersion than the CS method. A significant difference was found between the CS and the SC method (*p* = 0.01). For the slope SDs, smaller medians and narrower dispersions were found with RS and CS methods for all numbers of trials (Figure 13c). Significant differences were found in the distribution from the RS method (*p* = 0.04) and the CS method (*p* = 0.02) compared with the SC method when the number of trials was 15. When the data were combined (Figure 13d), significant differences were found between the RS and the SC method and between the CS and the SC method (both *p* < 0.01).

## 4. Discussion

The initial simulations illustrated that our random step (RS) algorithm generates a distribution of trials that follows a Gaussian distribution with an offset. This allows robust estimates of both PSEs and slopes, particularly when noise levels are high. These observations also transfer well to actual psychophysical experiments, in which confident estimates of the PSE and slope could be made with small numbers of trials. The PSE estimates obtained with both RS and constant stimuli (CS) correlate well together and show comparable variance.

The three methods we compared employ different strategies to map psychometric functions, as can be seen in the histograms of the test levels resulting from simulations (Figure 4b) and actual experiments (Figure 8). The CS method arranges the same number of trials at each level. The SC method clusters almost all the trials near the PSE, while very few trials are distributed in distant areas. And the RS method tends to have a performance in between. In the RS method, all trials are tested even at boundaries, but about 2 times more trials concentrate near the PSE estimate. It is particularly interesting to observe that the RS method performs similarly regardless of the number of trials in actual experiments. The peaks of the histogram have also moved as a function of the PSE in the left and right panels of Figure 8. Although, contrary to our expectations, no such obvious bump was found in the middle panel. This is probably because of the small number of trials we used and because the bump is less likely to be visible when it is in the middle of the range compared to the edges. Indeed, our method, by its design, will distribute approximately the same number of trials on both sides of the PSE. Therefore, when the PSE is close to an edge, there is on one side of it a narrower “buffer area”, which will concentrate a lot of trials and hence strongly contribute to the formation of the peak. 

This distribution presents two experimental advantages: (i) The fact that an even number of trials will be tested on both sides of the PSE will help prevent the behavioral bias of subjects from feeling they are responding up or down too often and might want to equalize their responses. And (ii) the fact that any level, even with large modulation (at the end of the range), can be tested anytime will prevent the subjects from becoming adapted to small modulations (close to their PSE) or feeling that the task becomes too complicated. 

In psychophysical experiments, the noise in the system can be characterized by the slope of the psychometric function. Noise, whether internal or external, can have dramatic effects on the quality of the estimation of the parameters of the psychometric function [2]. Therefore, we tested the constant stimuli, staircase, and adaptative methods against different levels of noise with simulations. We observed that increasing the amount of noise leads to more variable estimates of the PSE (Figure 6c) but more stable estimates of the slope (Figure 6d). This is reasonable because a higher noise level should directly result in a higher variance of the PSE and a shallower slope [29]. Our RS method was particularly robust at high noise levels with little variability and low failure rate, particularly compared to the SC method, whose distribution became much flatter than the other two as the noise level increased. We infer that at high noise levels, the SC method has difficulty pinpointing trials around the PSE estimates, as it can easily go in the wrong direction. This interference possibly makes it easy to be trapped around the wrong level and may lead to some inaccurate PSE estimates. In comparison, the RS method benefits from its widespread strategy. After each trial, the range in which the following level would be picked would still be big: anywhere between the current trial and one boundary of the range, allowing the RS method to sample the parameter space more evenly. And, considering very low noise, the PSE should theoretically always be in the newly picked range, thereby generating such bell shape distribution. In this case, the risk of missing the true PSE estimate will be reduced. 

Regarding the estimation of the slope, we observed that at high noise levels, the distribution of the SD of the estimates became sharper and shifted to the left (smaller mean) for all methods, as higher noise levels result in shallower slopes [27]. However, at low noise levels (σ=2), the SC method did outperform the other two methods. This was likely the consequence of the fact that, at low noise levels, the psychometric function would be very steep, and the RS and CS methods might not provide enough samples in the narrow effective range around the PSE. We also ran a control experiment with all three methods (RS, CS, and SC) for an even smaller number of trials (15, 30 and 45). Generally, all methods could gain a reasonable PSE estimate under all luminance conditions. As for the standard deviations, similar to what we found in simulations, the RS method showed no significant difference from the CS method but tended to be significantly different than the SC method. Overall, larger SDs of PSE and smaller SDs of slope were likely to be found in the RS method compared to the SC method. It turned out that the RS method kept a satisfying performance with a very small trial count.

Admittedly, there are still some limitations of our RS method. First, no evident bump in the distribution of trials could be found when the number of trials was small, and the PSE was located in the middle of the range of test levels (Figure 8, middle panel). Second, there are some cases where our RS method may fail to converge. For example, when the slope of the psychometric function is expected to be very steep (i.e., very low noise level), there would be a higher chance for the RS method (also the CS method) to be a convergence failure. Third, when confronted with an exceedingly small number of trials or instances of finger errors (mistakes made by subjects), the accuracy of PSE estimates using the RS method may be compromised. We conducted simulations of the RS method using two different trial quantities (45 and 300) and two lapse rates (0% and 5%). Based on the distribution of PSE estimates (see Supplementary Figure S1), experiments with a higher number of trials resulted in a more closely clustered distribution, indicating that increasing the number of trials enhances the precision of PSE estimates. It is important to note, however, that in specific scenarios, even shorter experiments can still produce meaningful and acceptable estimates. 

Psychophysical measurements investigate how the stimuli and the sensations of subjects are related by looking into the sensory threshold [2]. Abnormal thresholds or threshold shifts may help to reveal perceptual deficits in some patients. However, understanding and collaboration from subjects are required [30]. A long-period test is likely to lead to a decrement in the ability to concentrate and more biased performances [31]. Thus, short tests with good accuracy are needed, especially in clinical settings. Our novel method allocates trials to all the stimuli levels while rendering more weight to the levels around the PSE estimate. It provides robust estimates of both the PSE and slope under various noise levels and with small trial counts. 

## Data Availability

Data is available on request from the authors.

## Supplementary Materials

The following supporting information can be downloaded at: https://www.mdpi.com/article/10.3390/vision7040074/s1, Figure S1: Histograms of the probability density of PSE estimates from the Random Step (RS) method.
