# Supplementary material for: The Random Step Method for Measuring the Point of Subjective Equality
Source: Vision (Basel). 2023 Nov 15;7(4):74. doi: 10.3390/vision7040074 (PMC10661322; doi:10.3390/vision7040074)
Supplement: Supplementary file 1 [file vision-07-00074-s001.zip › vision-2556734-supplementary.pdf]

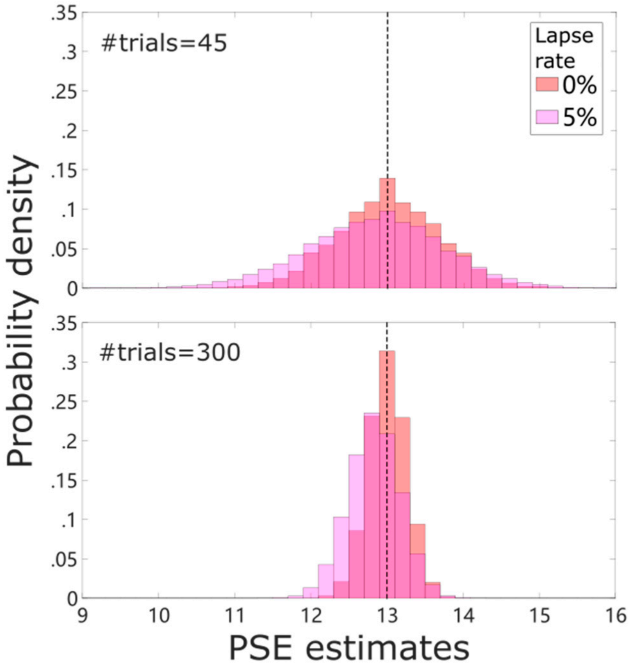

Supplementary Figure S1. Histograms of the probability density of PSE estimates from the Random Step (RS) method. The conditions simulated were 20 test levels and 10000 cycles. The comparison was employed between various trial amounts: 45 (top panel) and 300 (bottom panel); and lapse rates: 0% (red) and 5% (pink). The expected pivot was 13, marked out with a black dotted line.
